# Electronic Health Record–Nested Reminders for Serum Lithium Level Monitoring in Patients With Mood Disorder: Randomized Controlled Trial

**DOI:** 10.2196/40595

**Published:** 2023-03-22

**Authors:** Tomotsugu Seki, Morio Aki, Toshi A Furukawa, Hirotsugu Kawashima, Tomotaka Miki, Yujin Sawaki, Takaaki Ando, Kentaro Katsuragi, Takahiko Kawashima, Senkei Ueno, Takashi Miyagi, Shun'ichi Noma, Shiro Tanaka, Koji Kawakami

**Affiliations:** 1 Department of Pharmacoepidemiology School of Public Health, Graduate School of Medicine Kyoto University Kyoto Japan; 2 Department of Cardiovascular Medicine Graduate School of Medical Science Kyoto Prefectural University of Medicine Kyoto Japan; 3 Department of Psychiatry Toyooka Hospital Toyooka Japan; 4 Department of Psychiatry Kyoto University Hospital Kyoto Japan; 5 Department of Health Promotion and Human Behavior School of Public Health, Graduate School of Medicine Kyoto University Kyoto Japan; 6 National Epilepsy Center National Hospital Organization Shizuoka Institute of Epilepsy and Neurological Disorders Shizuoka Japan; 7 Department of Psychiatry Kyoto-Katsura Hospital Kyoto Japan; 8 Noma-Kokoro Clinic Kyoto Japan; 9 Department of Clinical Biostatistics Graduate School of Medicine Kyoto University Kyoto Japan

**Keywords:** electronic health record, lithium, mood disorders, randomized controlled trial, quality of health care

## Abstract

**Background:**

Clinical guidelines recommend regular serum lithium monitoring every 3 to 6 months. However, in the real world, only a minority of patients receive adequate monitoring.

**Objective:**

This study aims to examine whether the use of the electronic health record (EHR)–nested reminder system for serum lithium monitoring can help achieve serum lithium concentrations within the therapeutic range for patients on lithium maintenance therapy.

**Methods:**

We conducted an unblinded, single-center, EHR-nested, parallel-group, superiority randomized controlled trial comparing EHR-nested reminders with usual care in adult patients receiving lithium maintenance therapy for mood disorders. The primary outcome was the achievement of therapeutically appropriate serum lithium levels between 0.4 and 1.0 mEq/L at 18 months after enrollment. The key secondary outcomes are included as follows: the number of serum lithium level monitoring except for the first and final monitoring; exacerbation of the mood disorder during the study period, defined by hospitalization, increase in lithium dose, addition of antipsychotic drugs or mood stabilizers, or addition or increase of antidepressants; adherence defined by the proportion of days covered by lithium carbonate prescription during the study period.

**Results:**

A total of 111 patients were enrolled in this study. A total of 56 patients were assigned to the reminder group, and 55 patients were assigned to the usual care group. At the follow-up, 38 (69.1%) patients in the reminder group and 33 (60.0%) patients in the usual care group achieved the primary outcome (odds ratio 2.14, 95% CI 0.82-5.58, *P*=.12). The median number of serum lithium monitoring was 2 in the reminder group and 0 in the usual care group (rate ratio 3.62; 95% CI 2.47-5.29, *P*<.001). The exacerbation of mood disorders occurred in 17 (31.5%) patients in the reminder group and in 16 (34.8%) patients in the usual care group (odds ratio 0.97, 95% CI 0.42-2.28, *P*=.95).

**Conclusions:**

We found insufficient evidence for an EHR-nested reminder to increase the achievement of therapeutic serum lithium concentrations. However, the number of monitoring increased with relatively simple and inexpensive intervention. The EHR-based reminders may be useful to improve quality of care for patients on lithium maintenance therapy, and they have potentials to be applied to other problems.

**Trial Registration:**

University Hospital Medical Information Network Clinical Trials Registry UMIN000033633; https://tinyurl.com/5n7wtyav

## Introduction

Lithium carbonate is the most effective mood stabilizer in patients with bipolar disorder. It prevents both manic and depressive episodes and is an augmenting agent for recurrent or treatment-resistant major depressive disorder [1-3]. Owing to its narrow therapeutic range and the risks of renal dysfunction and hypothyroidism, guidelines strongly recommend regular serum lithium monitoring every week in the acute phase and every 3 to 6 months during the subsequent maintenance phase [4-8]. However, in Japan, only 14.9% of patients received serum lithium monitoring once a year or more between 2005 and 2014, whereas 30% to 65% of patients received monitoring at least once every 3 months in Europe [9-11].

Previous studies have shown that quality improvement programs such as active monitoring and reminder systems increase the serum lithium monitoring rate [12,13]. Recently, electronic health record (EHR)–nested alerts have been shown to improve quality of care [14,15]. However, no study has tested EHR-nested alerts for lithium monitoring.

Therefore, we mounted the Kyoto-Toyooka nested controlled trial of reminders (KONOTORI) trial to examine whether the use of the EHR-nested reminder system for serum lithium monitoring can help achieve serum lithium concentrations within the therapeutic range for patients on lithium maintenance therapy.

## Methods

### Trial Design

The KONOTORI trial was an unblinded, single-center, EHR-nested, parallel-group, superiority randomized controlled trial (RCT). This trial was conducted at the Department of Psychiatry of Toyooka Hospital, a tertiary-care community hospital in Toyooka City, Hyogo, Japan. The details of the trial design have been previously published (University Hospital Medical Information Network Clinical Trials Registry: UMIN000033633) [16].

All procedures contributing to this work were compliant with the ethical standards of the relevant national and institutional committees on human experimentation and the Helsinki Declaration of 1975, as revised in 2008. The study protocol was approved by the Ethics Committee of Kyoto University Graduate School of Medicine and the Institutional Review Board of Toyooka Hospital (C1401). All patients provided written informed consent prior to enrollment. This study followed the Consolidated Standards of Reporting Trials statement [17].

### Patient Selection and Randomization

Patients aged 18 years or older who were diagnosed with recurrent major depression, bipolar I disorder, or bipolar II disorder in accordance with the *Diagnostic and Statistical Manual of Mental Disorders*, 5th edition, and had been taking lithium carbonate for 6 months or longer were eligible for enrollment. The major exclusion criteria were as follows: prescribed lithium carbonate for reasons other than mood disorders, primary diagnosis of schizophrenia, suspected lithium intoxication, and contraindications to lithium carbonate. Multimedia Appendix 1 presents the full eligibility criteria. Eligible patients were randomly assigned in a 1:1 ratio to either the EHR-nested reminder or the usual care group. Randomization was performed with the use of the EHR-nested trial program, which was stratified according to the diagnosis based on the Primary Care Evaluation of Mental Disorders for major depression, bipolar I disorder, or bipolar II disorder and the baseline serum lithium levels (between 0.4 mEq/L and 1.0 mEq/L, <0.4 mEq/L, or >1.0 mEq/L), using permutated block randomization. An independent trial statistician generated the random allocation sequence. Randomization was performed at the patient’s first scheduled visit, 4 to 8 months after enrollment in the EHR system. Therefore, the allocation was concealed from the study personnel (psychiatrists and nurses) who enrolled patients.

### Trial Procedures and Interventions

**Figure 1:**
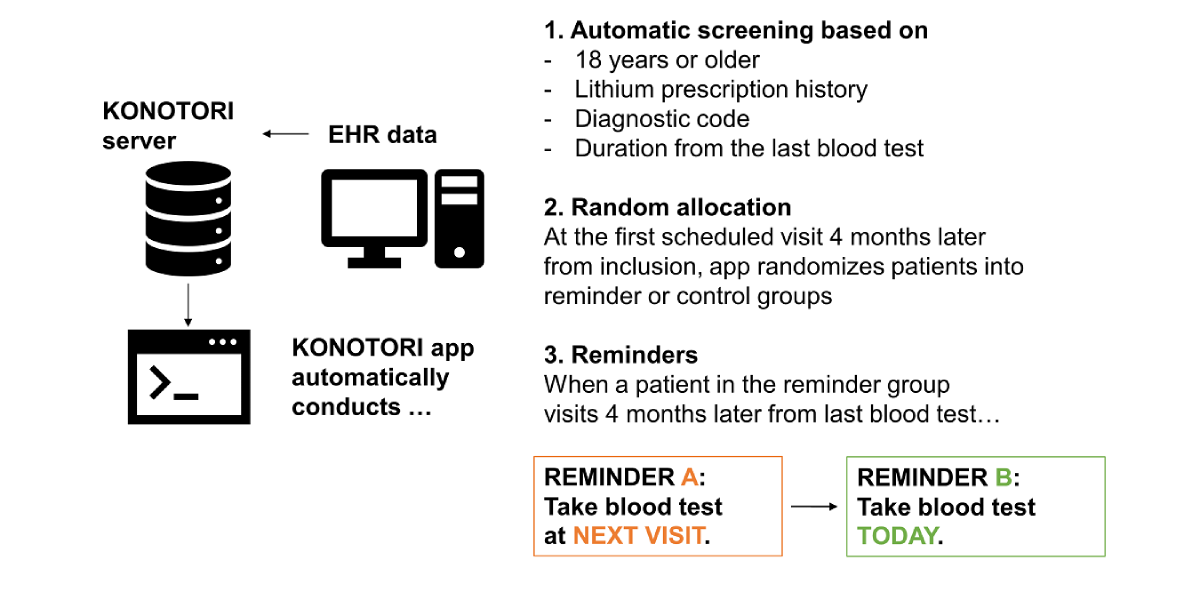
Illustration of KONOTORI system. EHR: electronic health record; KONOTORI: Kyoto-Toyooka nested controlled trial of reminders.

The trial program in the EHR system automatically prompted eligibility screening when potential candidates visited the hospital and also conducted the random allocation for the consenting patients at the prespecified timing above. When the patient was enrolled and allocated to the intervention arm, the trial program printed out reminders of serum lithium monitoring to the physician, as specified by the algorithm. When a patient in the reminder group visited 4 to 8 months after the last lithium monitoring or study registration, the first reminder was sent to the physician. This reminder included the following sentence: “Please notify the patient of the serum lithium monitoring at the next visit.” When the patient visited within 8 months of the first reminder, the second reminder was sent. The second reminder included the following sentence: “Please notify the patient of the serum lithium monitoring today.” These 2-step reminders were repeated up to 2 times. When patients were allocated to the usual care group, they received the usual care without reminders. No concurrent treatments were prohibited in this trial (Figure 1).

### Trial Outcomes

The primary outcome was the achievement of therapeutic serum lithium levels >0.4 but <1.0 mEq/L at the follow-up visit 18 months after enrollment [7]. If the final serum lithium level was not available, the patient was regarded as not having achieved the primary outcome. The prespecified secondary outcomes were as follows: the number of serum lithium level monitoring except for the first and final monitoring; exacerbation of the mood disorder during the study period, defined by hospitalization, increase in lithium dose, addition of antipsychotic drugs or mood stabilizers, or addition or increase of antidepressants; and adherence, defined by the proportion of days covered (PPDC) of lithium carbonate prescription during the study period. The safety outcomes included the estimated glomerular filtration rate (eGFR) and thyrotropin (TSH) levels at the follow-up visit. Data on serious adverse events, including death, were compiled for safety assessment.

### Sample Size Calculation

The baseline estimates were obtained from The Real World Data database (Health, Clinic, Education, Information Evaluation Institute/Real World Data, Co, Ltd), which includes approximately 19 million patients’ data from 170 institutions in Japan. According to this database, of 1464 patients who were prescribed lithium carbonate in a 6-month period up to June 1, 2018, therapeutic serum lithium levels (between 0.4 mEq/L and 1.0 mEq/L) were maintained in 818 (55.9%) patients. In this trial, we estimated that a total of 108 patients would provide 80% power if 48 (80%) patients in the reminder group and 33 (55%) patients in the usual care group achieved the primary outcome using the 2-sided chi-square test with a 2-side alpha level of .05. Assuming a dropout rate of 10%, the final sample size was set at 120.

### Statistical Analysis

All analyses were conducted in accordance with the intention-to-treat principle. We analyzed the primary outcome and exacerbation of mood disorders using logistic regression and reported the odds ratios (ORs) with *P* values and 95% CIs. The model included diagnosis and baseline serum lithium levels as covariates. We examined the number of serum lithium monitoring using Poisson regression, adjusted for the covariates listed above, and reported the rate ratios with their *P* values and 95% CIs. The PPDC of lithium carbonate prescription, eGFR, and TSH were analyzed using linear regression, adjusted with the same covariates, and reported with β coefficients, *P* values, and 95% CIs. In addition, as a post hoc analysis, the final serum lithium level was analyzed using linear regression as well. When the patient died or was lost to follow-up, the period between enrollment and the last visit was used as the denominator for the PPDC. To mitigate the potential bias caused by missing data, we conducted multiple imputation analyses under the assumption that the data were missing at random [18]. Multiple imputation was performed by creating 5 data sets using the fully conditional specification method and combining the effect estimates from each data set using the Rubin rule. All statistical tests were 2-sided, and *P* values <.05 were considered statistically significant. All analyses were conducted by academic researchers using SAS software, version 9.4 (SAS Institute).

## Results

### Overview

**Figure 2:**
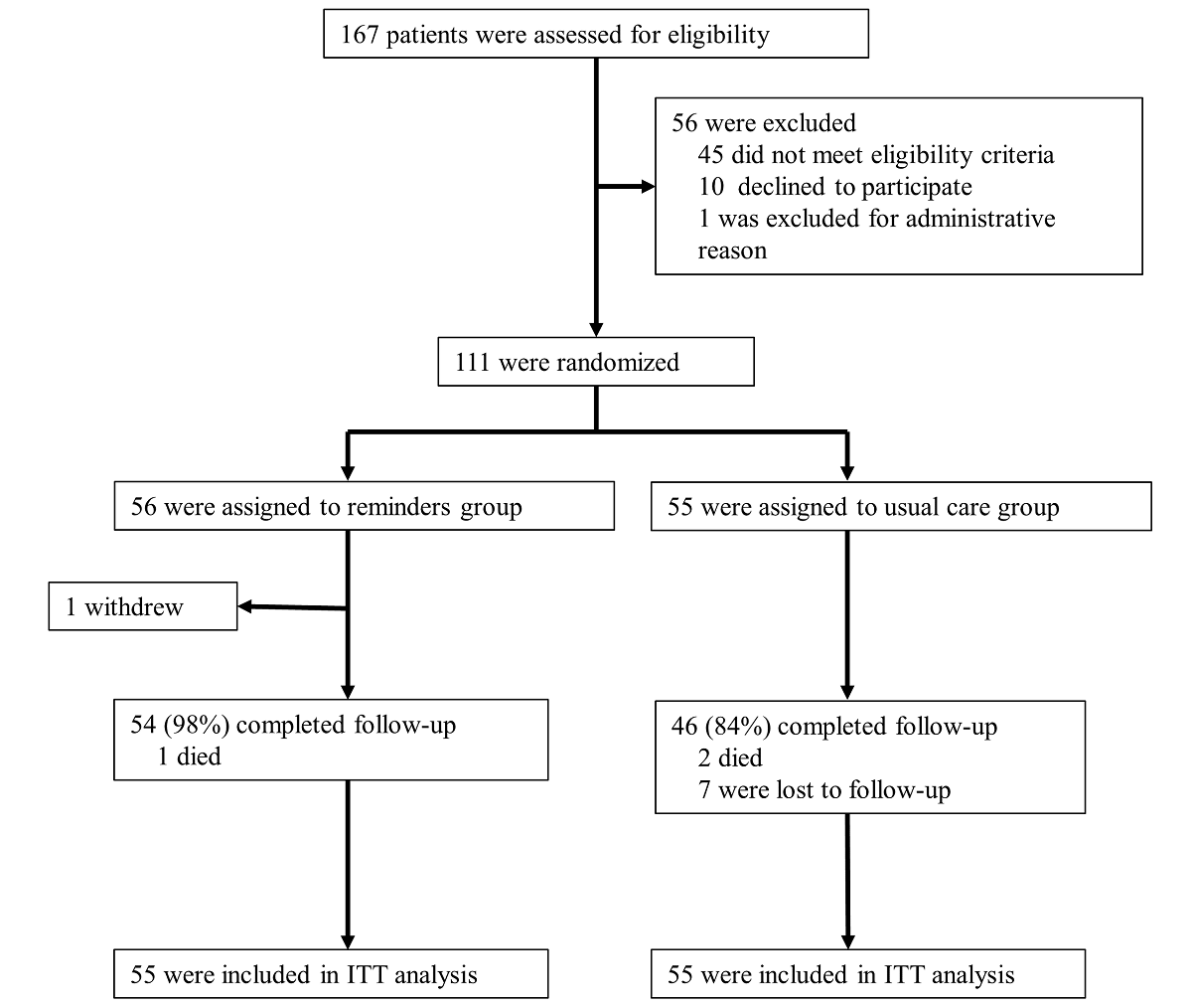
Study flow chart. ITT: intention to treat.

Trial enrollment started on November 1, 2018, and ended on March 31, 2020. We continued the follow-up until September 10, 2021. Of 167 patients who underwent screening, 56 were excluded (Figure 2) and 111 underwent randomization. Of them, 56 patients were randomly assigned to receive EHR-nested reminders (reminder group), and 55 received usual care (usual care group). One patient in the reminder group withdrew consent after randomization, and the remaining 110 patients were analyzed. The baseline characteristics were well balanced between the treatment groups (Table 1). A total of 48% of the patients had bipolar I disorder, 36% had bipolar II disorder, and 26% had recurrent major depression. The mean age of patients was 57 years, and 42.7% were men. The mean baseline lithium level was 0.61 mEq/L, and 73.6% of patients had lithium levels within the therapeutic range.

**Table 1:** Baseline characteristics of patients.

		Reminders group (N=55)	Usual care group (N=55)
Age (years), mean (SD)		58.2 (14.9)	55.7 (15.3)
Male sex, n (%)		24 (43.6)	23 (41.8)
**Diagnosis, n (%)**			
	Bipolar I disorder	23 (41.8)	25 (45.5)
	Bipolar II disorder	20 (36.4)	16 (29.1)
	Recurrent major depression	12 (21.8)	14 (25.5)
	Baseline lithium level (mEq/L)	0.62 (0.26)	0.60 (0.23)
**Baseline lithium level category, n (%)**			
	Normal (0.4-1.0 mEq/L)	39 (70.9)	42 (76.4)
	Low (<0.4 mEq/L)	11 (20.0)	11 (20.0)
	High (>1.0 mEq/L)	5 (9.1)	2 (3.6)
**Baseline lithium level (mEq/L), mean (SD)**			
	Overall	0.62 (0.26)	0.6 (0.23)
	Normal (0.4-1.0 mEq/L)	0.65 (0.14)	0.66 (0.15)
	Low (<0.4 mEq/L)	0.26 (0.1)	0.27 (0.14)
	High (>1.0 mEq/L)	1.11 (0.1)	1.06 (0.03)
**Laboratory data, mean (SD)**			
	Aspartate aminotransferase (mg/dL)	23.2 (8.4)	22.5 (8.7)
	Alanine aminotransferase (mg/dL)	23.2 (14.6)	25 (21.2)
	Serum creatinine level (mg/dL)	0.78 (0.17)	0.79 (0.19)
	eGFR^a^ (mL/min/1.73 m^2^)	71.2 (16.3)	71.2 (16.7)
**Concomitant drugs, n (%)**			
	Antipsychotics	30 (54.6)	33 (60.0)
	Antidepressants	15 (27.3)	15 (27.3)
	Mood stabilizers	25 (45.5)	26 (47.3)

^a^eGFR: estimated glomerular filtration rate.

### Clinical Efficacy Outcomes

At a median follow-up of 18 months, 54 (98%) patients in the reminder group and 46 (84%) patients in the usual care group completed the follow-up.

At the follow-up, 38 (69.1%) patients in the reminder group and 33 (60%) patients in the usual care group achieved therapeutic serum lithium levels, but the difference in the proportions was not significantly different between the 2 groups (adjusted OR 2.14, 95% CI 0.82-5.58, *P*=.12; risk difference 0.136, 95% CI -0.017 to 0.288, *P*=.08; risk ratio 1.17, 95% CI 0.94-1.47, *P*=.16). With multiple imputation, the result was consistent but more conservative than that of the primary analysis (adjusted OR 1.22, 95% CI 0.73-2.06, *P*=.45) (Table 2).

During this period, 94.4% of patients in the reminder group and 19.6% in the usual care group received serum lithium monitoring 2 times or more between the first and final tests (about once every 6 months; Table 3). The median number of serum lithium monitoring was 2 (IQR 2-3) in the intervention group and 0 (IQR 0-1) in the usual care group, and monitoring was more frequent in the intervention group (rate ratio 3.62, 95% CI, 2.47-5.29, *P*<.001).

Exacerbation of mood disorders occurred in 17 (31.5%) patients in the intervention group and 16 (34.8%) patients in the usual care group, and the proportion was not significantly different (OR 0.97, 95% CI 0.42-2.28, *P*=.95). The adherence reported by the PPDC was not significantly different between the 2 groups (Table 2).

**Table 2:** Primary and secondary outcomes.

		Reminders group (N=55)	Usual care group (N=55)	Effect size (95% CI)	*P* value
**Primary outcome^a^**					
	Achievement of therapeutic serum lithium level, n (%)	38 (69.1)	33 (60.0)	2.14 (0.82 to 5.58)	.12
**Secondary outcomes**					
	Number of serum lithium monitoring, median (IQR)^b^	2 (2-3)	0 (0-1)	3.62 (2.47 to 5.29)	<.001
	Exacerbation of mood disorders, n (%)^a^	17 (31.5)	16 (34.8)	0.97 (0.42 to 2.28)	.95
	Proportion of days covered, median (IQR)^c^	1.0 (0.99-1)	1.0 (0.99-1)	–0.016 (–0.046 to 0.014)	.28
	eGFR^d^ level, mean (SD)^c^	71.6 (20)	69.8 (16.4)	1.80 (–5.36 to 8.96)	.62
	Thyrotropin level, mean (SD)^c^	2.61 (2.66)	2.46 (1.62)	0.167 (–0.71 to 1.05)	.71
	Serum lithium level, mean (SD)^c^	0.57 (0.25)	0.56 (0.23)	0.026 (–0.058 to 0.109)	.55

^a^The effect sizes of the primary outcome and exacerbation of mood disorders are expressed as odds ratios with 95% CIs.

^b^The effect size of the number of serum lithium monitoring is expressed as a rate ratio with 95% CI.

^c^The effect sizes of the proportion of days covered, eGFR level, and thyrotropin level are expressed as β-coefficients with 95% CIs.

^d^eGFR: estimated glomerular filtration rate.

**Table 3:** Number of serum lithium monitoring.

Number of monitoring	Reminders group (N=54), n (%)	Usual care group (N=46), n (%)
None	0 (0)	24 (52.2)
1 time	3 (5.6)	13 (28.3)
2 times	27 (50)	7 (15.2)
3 times	18 (33.3)	2 (4.4)
4 or more times	6 (11.1)	0 (0)

### Safety Outcomes

The mean serum eGFR and TSH levels were similar between the 2 groups (Table 2). One patient in the intervention group discontinued lithium carbonate owing to lithium intoxication, and 1 patient in the usual care group temporarily discontinued but restarted it thereafter. One patient in the intervention group and 2 patients in the usual care group died for reasons unrelated to mood disorders, including suicide. Multimedia Appendix 2 summarizes the causes of death and loss to follow-up.

## Discussion

### Overview

In this single-center, unblinded EHR-nested RCT, we compared the effectiveness of EHR-nested reminders for serum lithium monitoring with that of usual care in patients with bipolar disorder or recurrent major depression. EHR-based reminders did not increase the achievement of therapeutic serum lithium concentration between 0.4 and 1.0 mEq/L at the follow-up visit compared to usual care. EHR-based reminders increased the number of serum lithium monitoring but did not decrease the exacerbation of mood disorders during the study period.

Monitoring rates of serum lithium level vary greatly among countries and have been reported to be relatively low in the United States and Japan. In the United States, one study showed that only 19% of patients received serum lithium monitoring within 6 months of initiation [19]. Another study showed that 36.5% of patients did not receive serum lithium monitoring at least once per year [20]. Moreover, in Japan, only 14.9% of patients receive serum lithium monitoring at least once a year [9]. Conversely, in Europe, 30% to 65% of patients receive serum lithium monitoring once every 3 months [10,11].

Therefore, further efforts to increase the serum lithium monitoring rate are needed when the monitoring rates are low. In the United Kingdom, audit-based quality improvement programs increase the number of patients with 4 serum lithium tests per year from 30% to 48% [12]. Similarly, active monitoring and reminder using the registry increased the proportion of patients who received serum lithium monitoring more than or equal to 4 times per year from 32.8% to 68.5% in Norfolk, United Kingdom [13]. Although these methods are certainly effective, they are also burdensome and costly.

In our study, the serum lithium monitoring rate dramatically increased to 94.4%, 3.6 times higher in the reminder group than the usual care group, with simple and inexpensive intervention. In addition, our intervention did not cause “EHR fatigue,” the phenomenon that physicians may stop responding to EHR-alerts when they are exposed to too many alerts [21]. Our EHR-based reminders may be implemented for other conditions, such as abnormal involuntary movement scale for tardive dyskinesia, while monitoring if EHR fatigue is not taking place. However, the large increase in monitoring rate may have been partly due to the study environment. As noted earlier, this study was conducted at a tertiary-care community hospital. Adherence to reminders may have been different if the study had been conducted in different environments, such as general practitioners’ offices, university hospitals, or multicenter trials.

Some studies have implemented and reported EHR-nested alerts. One study showed that EHR-nested alerts reduced unnecessary telemetry without worsening patient outcomes [14]. An EHR-based “pop-up” alert for acute kidney disease has increased the frequency of various care practices for acute kidney injury [15]. Recently, some RCTs, called EHR-nested RCTs, have used EHRs in their implementation [22]. EHR-nested RCTs aim to increase the feasibility of RCTs by using EHR to reduce trial costs, time burden, and human resources. Additionally, EHR-nested systems have increased the referral and recruitment of participants in some studies. However, no EHR-nested system has been shown to improve patient outcomes. In our study, the EHR-nested reminders did not increase the achievement of therapeutic serum concentration of lithium or decrease the exacerbation of mood disorders.

### Study Limitations

Our study has several limitations. First, caution is needed when interpreting the results. The primary outcome, the achievement of therapeutic serum lithium concentrations, was not statistically significant, but the OR of 2.14 was somewhat in favor of the EHR-nested alerts. However, more patients in the usual care group were lost to follow-up, mainly because of moving to another hospital, and these patients were regarded as not having achieved the primary outcome. Multiple imputation analyses showed a consistent, but more conservative, effect than primary analysis. Second, this study randomized individual patients rather than physicians. Physicians may have learned how often they should monitor serum lithium levels, and this would tend to bias the results toward the null that the alerts did not improve the primary outcome. However, this would have worked toward decreasing the difference, but in our study, the monitoring of serum lithium was significantly and clearly more frequent in the reminder group. Third, the study may have lacked the statistical power to detect the small effect of the alert system. Our planned sample size was 120; however, recruitment was stopped at 110 because the eligible patient pool at Toyooka Hospital was exhausted. Fourth, adherence in our study, which was measured by PPDC and reached nearly 100% in both groups, was quite higher than in the previous reports, which reported that the median adherence was 50% to 60% [23]. The excellent adherence in our patients was desirable but may have caused “ceiling effect,” which prevented us from detecting small differences. Finally, the generalizability of the findings of our study is limited because it was conducted in a rural tertiary-care hospital in Japan.

### Conclusions

In this single-center, unblinded, EHR-nested RCT, we found insufficient evidence for EHR-nested reminders to increase the achievement of therapeutic serum lithium concentration at 18 months after enrollment. However, the number of monitoring increased with a relatively simple and inexpensive intervention. The EHR-based reminders may be useful to improve quality of care for patients on lithium maintenance therapy, and they have potential to be applied to other problems.

## Appendix

Multimedia Appendix 1 Eligibility criteria.

Multimedia Appendix 2 Reasons of death and unable to follow-up.

Multimedia Appendix 3 CONSORT-eHEALTH checklist (V 1.6.1).

## Abbreviations

eGFR: estimated glomerular filtration rate
EHR: electronic health record
KONOTORI: Kyoto-Toyooka nested controlled trial of reminders
PPDC: proportion of days covered
RCT: randomized controlled trial
OR: odds ratio
TSH: thyrotropin
