# Correction: Electronic Health Record–Nested Reminders for Serum Lithium Level Monitoring in Patients With Mood Disorder: Randomized Controlled Trial

**DOI:** 10.2196/55805

**Published:** 2024-01-03

**Authors:** Tomotsugu Seki, Morio Aki, Toshi A Furukawa, Hirotsugu Kawashima, Tomotaka Miki, Yujin Sawaki, Takaaki Ando, Kentaro Katsuragi, Takahiko Kawashima, Senkei Ueno, Takashi Miyagi, Shun'ichi Noma, Shiro Tanaka, Koji Kawakami

**Affiliations:** 1 Department of Pharmacoepidemiology School of Public Health, Graduate School of Medicine Kyoto University Kyoto Japan; 2 Department of Cardiovascular Medicine Graduate School of Medical Science Kyoto Prefectural University of Medicine Kyoto Japan; 3 Department of Psychiatry Toyooka Hospital Toyooka Japan; 4 Department of Psychiatry Kyoto University Hospital Kyoto Japan; 5 Department of Health Promotion and Human Behavior School of Public Health, Graduate School of Medicine Kyoto University Kyoto Japan; 6 National Epilepsy Center National Hospital Organization Shizuoka Institute of Epilepsy and Neurological Disorders Shizuoka Japan; 7 Department of Psychiatry Kyoto-Katsura Hospital Kyoto Japan; 8 Noma-Kokoro Clinic Kyoto Japan; 9 Department of Clinical Biostatistics Graduate School of Medicine Kyoto University Kyoto Japan

The authors of “Electronic Health Record–Nested Reminders for Serum Lithium Level Monitoring in Patients With Mood Disorder: Randomized Controlled Trial” (J Med Internet Res 2023;25:e40595) noted an error in the *Sample Size Calculation* subsection of the *Methods* section.

Originally, the power in the sample size calculation was reported as “90%,” but the correct power for the study was “80%.”

The sentence

In this trial, we estimated that a total of 108 patients would provide 90% power if 48 (80%) patients in the reminder group and 33 (55%) patients in the usual care group achieved the primary outcome using the 2-sided chi-square test with a 2-side alpha level of .05. Assuming a dropout rate of 10%, the final sample size was set at 120.

was changed to

In this trial, we estimated that a total of 108 patients would provide 80% power if 48 (80%) patients in the reminder group and 33 (55%) patients in the usual care group achieved the primary outcome using the 2-sided chi-square test with a 2-side alpha level of .05. Assuming a dropout rate of 10%, the final sample size was set at 120.

This error does not affect the study's methodology, results, or conclusions, but it is important to correct the record for accuracy and clarity.

The correction will appear in the online version of the paper on the JMIR Publications website on January 3, 2024 together with the publication of this correction notice. Because this was made after submission to PubMed, PubMed Central, and other full-text repositories, the corrected article has also been resubmitted to those repositories.

