# Supplementary material for: Electronic Health Record–Nested Reminders for Serum Lithium Level Monitoring in Patients With Mood Disorder: Randomized Controlled Trial
Source: J Med Internet Res. 2023 Mar 22;25:e40595. doi: 10.2196/40595 (PMC10139684; doi:10.2196/40595)
Supplement: Multimedia Appendix 1 [file jmir_v25i1e40595_app1.docx]

Multimedia Appendix 1. Eligibility criteria

Participants will be recruited in accordance with the eligibility criteria described below.

Inclusion criteria

The participant must fulfill all the following:

1. Age ≥ 18 years on the day of informed consent

2. Has recurrent major depression, bipolar I disorder, or bipolar II disorder according to the Diagnostic and statistical manual of mental disorders, 5th edition (DSM-5)

3. Has been taking lithium carbonate for 6 months or longer

4. Has been judged by the treating physician to need a prescription of lithium carbonate for the next 18 months

Exclusion criteria

The participant must not meet any of the following criteria:

1. Prescribed lithium carbonate for an indication other than mood disorders

2. A primary diagnosis of schizophrenia

3. Judged by the treating physician to have an imminent high risk of suicide

4. Suspected to have lithium intoxication

5. Women who are pregnant or breastfeeding

6. Cohabiting family members of study staff personnel

7. Inability to understand written Japanese

8. Contraindications to lithium carbonate

9. Participating in another clinical trial

10. Currently hospitalized

11. Terminal physical disease

12. No serum lithium concentration available within 7 days of informed consent

13. No appointment between 4 and 8 months after informed consent

14. Written informed consent is unavailable

15. Judged by the treating physician as inappropriate for participation

(criteria 12 and 13 may be confirmed after informed consent, but before randomization)
