# Supplementary material for: Electronic Health Record–Nested Reminders for Serum Lithium Level Monitoring in Patients With Mood Disorder: Randomized Controlled Trial
Source: J Med Internet Res. 2023 Mar 22;25:e40595. doi: 10.2196/40595 (PMC10139684; doi:10.2196/40595)
Supplement: Multimedia Appendix 2 [file jmir_v25i1e40595_app2.docx]

Multimedia Appendix 2. Reasons of death and unable to follow-up

| **Reasons of death** | n |
| --- | --- |
| Accident (drowning) | 1 |
| Myocardial infarction | 1 |
| Pneumonia | 1 |
| **Reasons of unable to follow-up** |  |
| Moving to another hospital | 5 |
| Telephone visit due to COVID-19 | 1 |
| Unknown | 1 |
